# Successful Formulation and Application of Plant Growth-Promoting* Kosakonia radicincitans* in Maize Cultivation

**DOI:** 10.1155/2018/6439481

**Published:** 2018-03-28

**Authors:** Beatrice Berger, Sascha Patz, Silke Ruppel, Kristin Dietel, Sebastian Faetke, Helmut Junge, Matthias Becker

**Affiliations:** ^1^Leibniz Institute of Vegetable and Ornamental Crops, Grossbeeren, Germany; ^2^Institute for National and International Plant Health, Julius Kühn-Institute, Federal Research Centre for Cultivated Plants, Braunschweig, Germany; ^3^Algorithms in Bioinformatics, Center for Bioinformatics, University of Tuebingen, Tuebingen, Germany; ^4^ABiTEP GmbH, Berlin, Germany

## Abstract

The global market for biosupplements is expected to grow by 14 percent between 2014 and 2019 as a consequence of the proven benefits of biosupplements on crop yields, soil fertility, and fertilizer efficiency. One important segment of biosupplements is plant growth-promoting bacteria (PGPB). Although many potential PGPB have been discovered, suitable biotechnological processing and shelf-life stability of the bacteria are challenges to overcome for their successful use as biosupplements. Here, the plant growth-promoting Gram-negative strain* Kosakonia radicincitans* DSM 16656^T^ (family Enterobacteriaceae) was biotechnologically processed and applied in the field. Solid or liquid formulations of* K. radicincitans* were diluted in water and sprayed on young maize plants (*Zea mays *L.). Shelf-life stability tests of formulated bacteria were performed under 4°C and −20°C storage conditions. In parallel, the bacterial formulations were tested at three different farm level field plots characterized by different soil properties. Maize yield was recorded at harvest time, and both formulations increased maize yields in silage as well as grain maize, underlining their positive impact on different agricultural systems. Our results demonstrate that bacteria of the family* Enterobacteriaceae,* although incapable of forming spores, can be processed to successful biosupplements.

## 1. Introduction

Within the next 40 years agricultural production must increase by 60% to meet worldwide demands for food. However, arable land will only increase by five percent by 2050, and to date 25% of the arable land is already severely degraded. Both increasing food demands and diminishing arable land call for strategies to intensify agricultural systems without harming the environment. Maize, rice, and wheat provide at least 30% of food calories to more than 4.5 billion people in developing countries [1].

Maize mainly serves as fodder for livestock, where maize silage is an important forage component for ruminants, especially dairy cows. A minor portion of silage maize, 0.8 billion hectares, is used for producing ethanol and biogas. Grain maize serves as forage for pig fattening in stock farming. Since maize contains high amounts of starch, it is also of particular interest to the food industry as maize meal in human food, as well as for the paper producing industry.

In 2016, the total land area used for maize cultivation in Germany was 4.163 million hectares, and a total of 4,017,800 tonnes of maize was produced in Germany [2].

Maize cultivation is highly nutrient demanding, particularly for mineral nitrogen such as nitrate and ammonia. However, synthetic production (and field application) of mineral nitrogen is cost- and energy-intensive. Complementary procedure such as using biological atmospheric nitrogen fixing leguminous crops like beans or clover in crop rotation is one strategy to provide additional nitrogen to plants. Another strategy is the application of organic fertilizers as manure or slurry. Both strategies require microorganisms that convert atmospheric nitrogen or organic nitrogen into mineral nitrogen. Drawbacks associated with organic fertilization include significant nitrogen losses through ammonia volatilization, nitrates leaching into groundwater, and denitrification, as reviewed by Cameron et al. [3].

The application of plant-supporting microorganisms such as arbuscular mycorrhizal (AM) fungi, other fungi, and plant growth-promoting bacteria (PGPB) offers an attractive alternative strategy [4–7], since their application increases crop yields without adding additional mineral nitrogen. In maize, studies have shown how the application of microorganisms contributes to the growth of plants [8–12]. Among the microorganisms that promote growth and yield of maize is the Gram-negative, rod shaped bacterium* Kosakonia radicincitans* from the family of Enterobacteriaceae [13, 14].

In previous studies we tested* K. radicincitans* strain DSM 16656^T^, isolated from the phyllosphere of wheat, for its plant growth-promoting capacity.* In vitro* analyses showed that DSM 16656^T^ is able to fix atmospheric nitrogen [15, 16] and solubilize rock phosphates [17]. Moreover, this strain produces phytohormones as auxin and cytokine-like compounds [18]. The* in vitro* characterization of DSM 16656^T^ was followed by several glasshouse and field experiments where seeds or young plants were inoculated with the strain at various concentrations to assess the most appropriate way and amount to exploit the plant growth-promoting effect of* K. radicincitans*. Besides maize, among the species that responded positively to inoculation with* K. radicincitans* we identified wheat [19], tomato [20], pea [21], and different members of the cabbage family [22, 23]. Significant increases in growth and yield promoted by* K. radicincitans* in greenhouse and field trials were confirmed [14, 15, 20, 22, 24], highlighting the potential of this strain for benefitting different cultivation management systems.

The successful transfer of biological supplements based on living microorganisms such as* K. radicincitans* DSM 16656^T^ from controlled greenhouse pot experiments to field cultivation approval is highly challenging. Problems are due to the huge variability not only in natural soil parameters, such as composition, graininess, water holding capacity and pH value, and the associated microbiota, but also in environmental conditions such as precipitation, air humidity, and temperature; all these factors interfere with the bacteria-plant interaction rendering the outcome of field experiments often difficult to predict.

Even more challenging is effective biotechnological processing and formulation of a potential strain like our* K. radicincitans* DSM 16656^T^ to transform it into a biosupplement suitable for application in agricultural systems. The biological supplement must be produced cost-efficiently, retain its positive traits during biotechnical processing steps, and be stable over a period of at least six months. Additionally, handling the product must be easy and the product must be robust enough for practical use in the field at the farm level. So far, biotechnological processing approaches include preparation of polymeric biodegradable low-cost foams [25], liquid formulation [26], or powders [27]. Still, it is a challenge to generate a robust biological supplement from Gram-negative bacteria. The inability of Gram-negative bacteria to form spores, as Gram-positive bacteria do under detrimental conditions, requires more sophisticated freeze-drying and biotechnological processing strategies. So far, successful formulation of Gram-negative bacteria is mostly described for* Pseudomonas* spp. [28, 29] and* Azospirillum* spp. [26], but not for* Kosakonia* spp. Ultimately, the final proof as to whether the bacterial formulation is sufficient to persist and have a successful impact on plant hosts still has to be tested in the field under real farm conditions [30].

Here, we present a study that describes the positive effects of* K. radicincitans* DSM 16656^T^ based biosupplement prototype, AbiVital®, on maize growth and yield in three different field plots in Germany.

## 2. Materials and Methods

### 2.1. Bacterial Strain Description and Biotechnological Processing

Aliquots of* K. radicincitans* DSM 16656^T^ were kept lyophilized at −80°C. For further cultivation bacteria were plated on ENDO agar and maintained at 4°C. Formulation of* K. radicincitans* was carried out by ABiTEP GmbH, Berlin. The product is listed as AbiVital soil auxiliary supplies in the German FiBL list, which regulates the implementation of microorganisms as resources in sustainable agriculture [31]. The AbiVital product comprises 64% (percent by weight) of centrifuged (7200 rpm)* K. radicincitans* DSM 16656^T^ cells from liquid culture and 36% cryoadditives. It contains less than 1.5% N, less than 0.5% P_2_O_5_, and less than 0.75% K_2_O to meet requirements of soil auxiliary supplies.

### 2.2. Shelf-Life Studies of Processed Bacteria

Formulations of solid and liquid AbiVital were tested for their shelf-life properties. Viable cell concentrations for each formulation were determined directly after fermentation by colony counting on agar plates according to ISO 4833-2 and in parallel performing an electrooptical analysis of bacterial cells using* EloTrace*® [32, 33].

Four biological replicates per formulation were analyzed subsequently for their shelf-life; independent subsamples were stored after the formulation process either at 4°C or at −20°C over a period of six months and monitored for viable cells using the most probable number (MPN) method, two, three, four, and six months after formulation. For the solid formulation, 1 g was weighed to an Eppendorf tube, ten clean glass pearls were added, and for the liquid formulation 1 mL was pipetted into an Eppendorf tube. Each formulation was transferred to a 50 mL Erlenmeyer flask containing a 9 mL standard nutrition broth for microbial cultivation (Merck, Germany). The flask was placed on a shaker at 200 rpm at 4°C for 1 h to dissociate the cells homogenously in the solution. The homogenous solution of each formulation was serially diluted by a factor of ten. The optical density as an indicator of the most probable number of viable cells was measured at 620 nm in a Tecan plate reader (Tecan, Germany). After adding 180 *μ*L of standard nutrient broth to each well of a microtiter plate, 20 *μ*L of the diluted formulation was added to the respective wells, incubated for 72 h at 30°C, and shaken for ten seconds just before measurement. Three dilutions of each sample were measured in two technical replicates. Wells containing 200 *μ*L of standard nutrient broth and no formulation served as a blank.

### 2.3. Gadsdorf 2015 and 2016 Field Experiments

Field experiments in Gadsdorf (soil type loamy sand) were conducted under conditions of organic farming.* Zea mays* var. P 7902 (Pioneer Hi Breed, Buxtehude, Germany) was sown on April 28, 2015. Two different formulations, solid (1) and liquid (2), were tested in Gadsdorf field trials for their impact on corn maize yield. Both formulations were added directly into the tank of the field spraying device (Amazone UX 5200 Super). Altogether, six hectares (approx. 570,000 plants) were sprayed on May 16, 2015, with formulation (1), formulation (2), or control liquid (=water) (2 hectares each). Additionally, formulations and control liquid were applied to neighboring subareas of the same field. Firstly, the control field site was sprayed with water. Secondly, the solid and liquid formulations of the bacterial strain* K. radicincitans* were diluted in water to a final concentration of 10^7^ cfu mL^−1^, and each plant received approximately 1 mL. Maize was harvested on November 2, 2015. The total weight of harvested kernels per subarea was determined.

### 2.4. Dannenberg 2015 and Sanitz 2016 Field Experiments

Field experiments in Danneberg and Sanitz were conducted to test the effect of the liquid formulation on silage maize grown in conventional cultivation systems. In 2015, a farm level field experiment was performed in Dannenberg, Lower Saxony. The plant cultivar was* Zea mays* var. Ronaldino (KWS, Germany); the soil was loamy sand. Plants were not treated with pesticides or fungicides. In contrast to field experiments in Gadsdorf, plant growth in Dannenberg and Sanitz was determined on smaller areas and then extrapolated to hectare sizes. Plant growth in Dannenberg was determined by harvesting subplots within the field from six randomly chosen spots (seven plants each) in the* Kosakonia*-treated section of the field and the control section of the field without bacterial application.

Consecutively, in 2016 the* Kürzinger GbR-agro nord *experimental station conducted an exact trial field experiment with* Zea mays* var. Colisee (KWS, Germany) on a loamy soil under good experimental practice (GEP) certificated conditions. Per hectare, 2.5 L of formulated AbiVital was sprayed. Plant growth was determined on four lots of 18 square meters per treatment.

### 2.5. Statistical Data Analysis

Data from field trials in Sanitz and Dannenberg were analyzed with SigmaPlot Version 12.3. Normality of data was tested by Shapiro-Wilk before using the *t*-test to compare the two treatments of noninoculated and AbiVital inoculated maize plants grown either in Sanitz or in Dannenberg. Experiments in Gadsdorf were performed in strip vials and analyzed with the adjusted mean value according to the guidelines of Michel and colleagues [34].

## 3. Results

### 3.1. Shelf-Life Studies on AbiVital Formulations

Shelf-life was tested by the most probable number method in four individual subsamples of formulated* K. radicincitans*. Bacterial viable counts in both solid and liquid formulations of* K. radicincitans* remained stable over the period of six months when they were stored at −20°C. In contrast, at 4°C only the solid formulation was stable during the 6-month period; viable counts in the liquid formulation decreased drastically during this time frame by >99% (Figure 2).

### 3.2. Silage Maize Treatment Performed in 2015/2016

We tested* K. radicincitans* in a liquid formulation of AbiVital in conventionally grown silage maize at two different plots in Germany, Dannenberg and Sanitz (Figure 3). In both plots, young maize plants were sprayed with the product. The total yield of noninoculated maize plants was 106.8 t per hectare in Sanitz and 107.8 t per hectare in Dannenberg, whereas the inoculation of maize plants with AbiVital resulted in 122.8 t per hectare in Sanitz (*t*-test, *P* = 0.002), which means a 14.9% increase in total weight and the yield increased even more in Dannenberg, by 29.3% = 139.4 t per hectare (*t*-test, *P* = 0.002) (Figure 3). Looking at cobs and aerial plant parts separately, we measured a cob yield increase of 32.2% (*t*-test, *P* = 0.048) from 35.4 t in control to 46.9 t in inoculated plants in Dannenberg and an 11.9% increase (*t*-test, *P* < 0.001) from 23.4 t in control to 26.2 t per hectare in Sanitz. The increase of aerial plant mass was 27.8% in Dannenberg (*t*-test, *P* < 0.001) and 15.8% in Sanitz (*t*-test, *P* = 0.008) (Figure 3).

### 3.3. Grain Corn Treatment

In a second approach we tested the formulated* K. radicincitans* product AbiVital on grain maize grown in organic cultivation management in Gadsdorf, Brandenburg, Germany. We tested two formulations, solid and liquid, in two consecutive years. In 2015, we found an increase in grain corn yield of 18.7% when using solid and an increase of 32.8% when using the liquid formulations of AbiVital. For the solid formulation, we obtained a similar effect of 20% increase in 2016, while the liquid formulation promoted an increase of 9.7% (Figure 4).

## 4. Discussion

Microorganisms represent a tremendous source of plant growth-promoting additives for application in agriculture. However, only a minority of potential microorganisms have been used in agriculture as yet. This is due either to the limited cultivation and isolation of bacteria from environmental samples [35], or to the failure to follow up processing towards a stable and efficient product [36]. Therefore, successful formulation under large-scale production conditions is crucial for commercial bacterial inoculants. Experiments in 1990–1992 already documented the positive effects of* K. radicincitans* DSM 16656^T^ on maize cv. Bekenova: grain yield increased by 8–15%, and shoot dry matter by 3–7% after inoculation with the bacteria [37].

Importantly, our results document for the first time the successful development of a bacterial isolate into a biosupplement for maize cultivation. The AbiVital formulation of the Gram-negative bacterium* K. radicincitans* preserves its plant growth-promoting properties, as shown in our field experiments with maize.

Differences in growth promotion were also described for a* Bacillus *sp. in two lima bean varieties [38], and inoculation with the same mycorrhiza on three rice ecotypes also resulted in different responses [39]. In* Phaseolus vulgaris*,* Azospirillum *spp. affects the* Rhizobium*-legume symbiosis, according to the plant's genotype [40]. However, we observed a positive promotion effect in all maize varieties tested with the newly formulated* K. radicincitans* DSM 16656^T^ biosupplement, suggesting no trade-offs in maize. The fact that maize was found to be the native host of a plant growth-promoting strain of* K. radicincitans* (GXGL-4A) [41] strongly supports the potential of this species as a biosupplement in maize cultivation. Several reports on plant growth-promoting* K. radicincitans* strains from different crops in different habitats around the world have been published in the past few years (Becker et al. submitted).

Among the factors that interfere with the effect of exogenously applied microorganisms on plants are soil composition and tillage management. According to ascertainments of the Federal Statistical Office in 2016, German farming is mostly conventional (92.8%), and only a minor part is organic farming (7.2%) [42]; but the latter is increasing since demands for organic farming products are growing rapidly. However, organic farming relies on strict guidelines. In general, conventional management systems allow not only more tillage than organic farming, but also the application of chemicals for weed and pest control. It is essential to know whether preprocessing, fertilization management, use of pesticides, or other differences between the farming systems would elevate or depress the effect of the growth-promoting bacteria before the commercialization of the “AbiVital” formulation. For instance, soil disturbances by tillage can cause qualitative and quantitative changes in soil microbiota and biological nitrogen fixation [43, 44]. Knowledge about how soil management changes microbial community structures is a prerequisite for optimized management practices, since soil microbial communities constitute a major factor controlling soil processes and plant growth [45–47].

**Figure 1:**
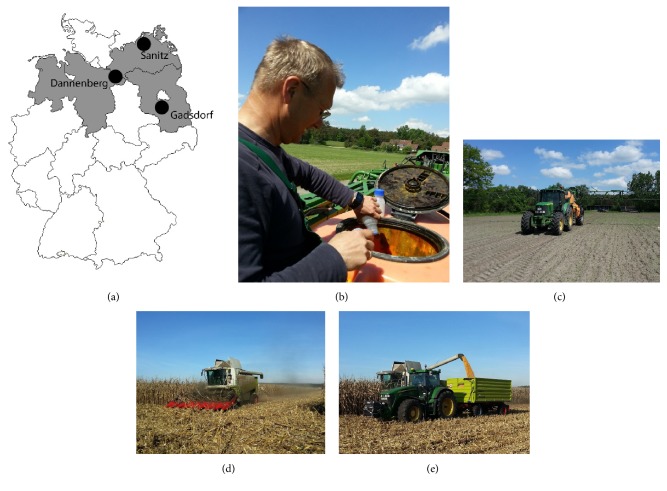
*Testing K. radicincitans formulations in conventionally grown silage maize.* (a) Map of Germany with field plots for maize experiments in Dannenberg (Lower Saxony), Sanitz (Mecklenburg-West Pomerania) and Gadsdorf (Brandenburg) in 2015 and 2016. (b) Preparation of formulated* Kosakonia radicincitans* for application on young maize plants. (c) Application of either liquid or solid bacterial formulation AbiVital by spraying it onto young maize plants when two leaves emerge. (d-e) Maize seeds harvested by combine harvester.

**Figure 2:**
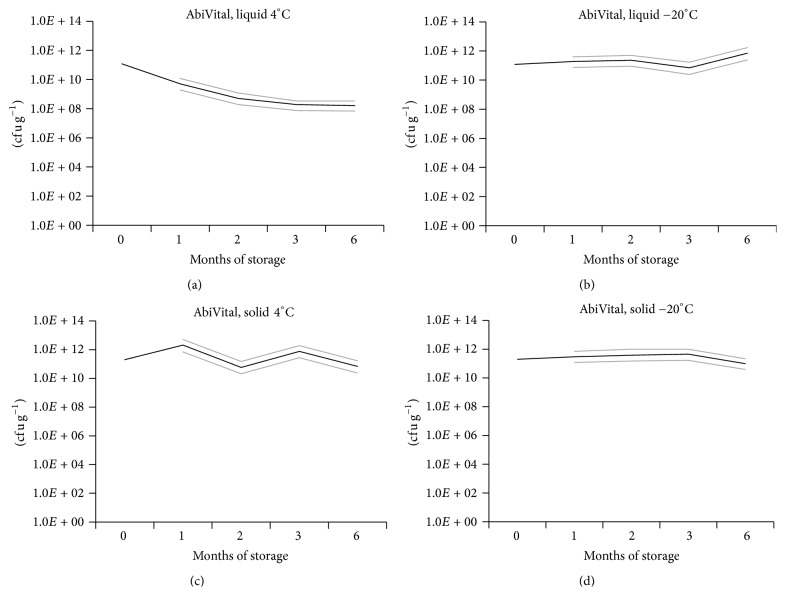
*Testing the shelf-life of AbiVital formulations.* Most probable number of vital* Kosakonia radicincitans* cells per mL in (a) AbiVital liquid, stored at 4°C; (b) AbiVital liquid, stored at −20°C, or cells per g in (c) AbiVital solid, stored at 4°C, and (d) AbiVital solid, stored at −20°C (black lines). Their respective low and high confidence intervals for 95% (grey lines) are shown after a period of 1, 2, 3, and 6 months of storage. *n* = 4.

**Figure 3:**
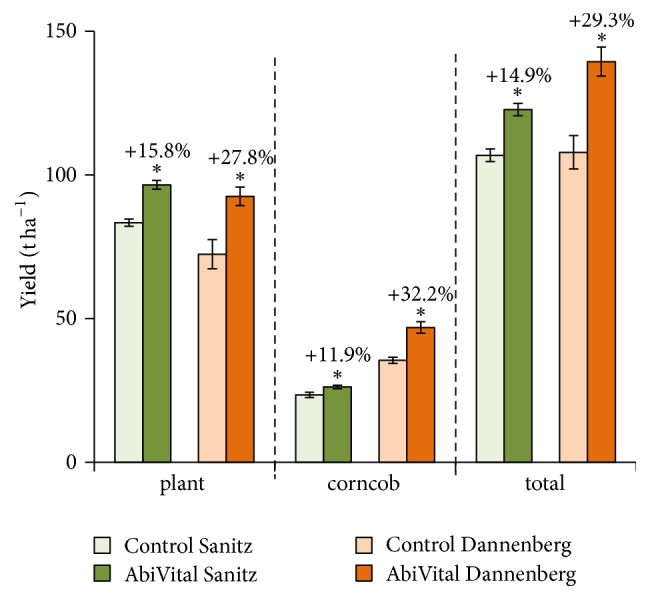
*Yield of silage maize in Sanitz (Mecklenburg-West Pomerania) and Dannenberg (Lower Saxony).* Plant weight, corn-cob weight, and total plant weight in maize plants either treated with AbiVital (dark bars) or in untreated control plants (light bars). Asterisks indicate statistical differences between inoculated plants and their respective controls, *t*-test ^*∗*^*P* > 0.05, *n* = 4 plotting replicates in Sanitz and *n* = 6 plotting replicates in Dannenberg.

**Figure 4:**
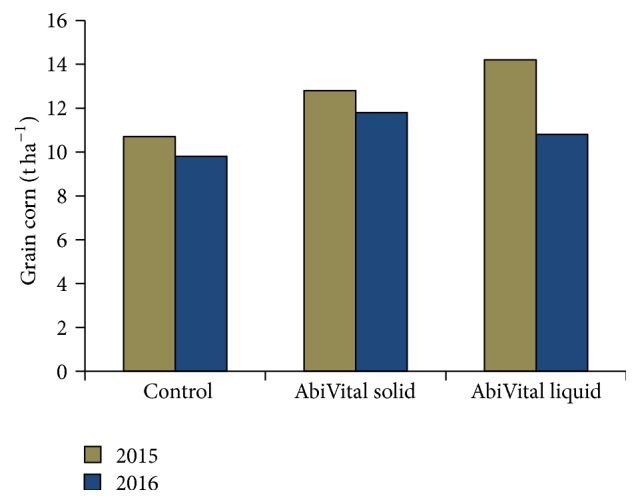
*The effect of formulated K. radicincitans AbiVital on grain maize grown in organic cultivation management.* Total grain corn production (t ha^−1^) in control, AbiVital solid and AbiVital liquid treated maize plants in 2015 (brown bars) and 2016 (blue bars) in Gadsdorf, Brandenburg.

To our knowledge, this is the first formulation and successful application of* Kosakonia *spp. in field grown maize plants. Liquid formulations are often preferred by the user because the product is easy to mix in a tank and cheaper to produce. Powder formulations are easier to transport and more stable, but the dry formulations must be easy to dissolve. The formulated carrier supplement plays an important role in delivering the bacteria to the field, and carriers can mainly be divided into the following categories: soils, inert material such as polymers or vermiculite, liquid formulation with additives, oil-dried bacteria, or just the plain lyophilized microbial culture. Biochar as an inoculant carrier has been proposed for developing new formulations [48]. Our product is free of genetically modified organism (GMO) carriers and complies with all economic and farm level application demands. It was shown to be easily manageable by the farmers in the field (Figure 1) and resulted in the same efficiency as previously used cultures produced in experimental laboratory conditions.

Rapid decrease in shelf-life for liquid products is a severe problem in the biotechnological processing of microorganisms for application in agriculture. A period of at least six months without drastic losses of vital cells is required in industrialized countries. During this period, loss-free storage should be achievable in already existing devices such as fridges or freezers. In developing countries the shelf-life requirements are even higher. Some studies claim a shelf-life of one or two years at room temperature [49, 50]. Our objective was to achieve shelf-life stability over six months at 4°C or −20°C with both formulations. Although the AbiVital biosupplement formulation shows promising results for storing at −20°C, further investigations will be needed into how to ensure a stable product during storage at 4°C.

Exhibiting clear growth and yield-promoting effects on crop plants, the use of microbial products is of interest in both conventional and organic farming systems. However, variable outcomes from applying microbial supplements have damaged their reputation as an environmentally friendly additive in agriculture. Advocates of conventional farming practices applying synthetic pesticides and microbial supplements being sold in ineffective concentrations have further contributed to the poor reputation of plant growth-promoting microorganisms. Nonetheless, the number of reliable microbial products on the market is increasing. To determine the benefits of microorganisms in crop farming and the circumstances under which they tap into their full potential, a combination of basic and applied research on the same strains of microorganisms is required. Deciphering the complex interactions of microorganisms, host plants and the environment will require interdisciplinary collaboration of botanists, microbiologists, biotechnologists, molecular biologists, bioinformaticians, and farmers.

## 5. Conclusions

We present the formulation of the Gram-negative bacterium* K. radicincitans* as a marketable product for application in silage and grain maize production. We show that the same bacterial strain is able to increase yields of silage and grain maize in conventional and organic farming at different sites in Germany. To generate a reliable product with a long shelf-life, many years of fundamental and applied research are needed. Today* K. radicincitans* DSM 16656^T^ is one of the best-studied PGPB and can be considered a model organism for research into beneficial bacteria from the family of Enterobacteriaceae. Developing a biosupplement from this strain is the outcome of 30 years of* Kosakonia* research, where DSM 16656^T^ proved repeatedly to have a beneficial impact on plant growth and yield.
